# Emerging Trends in Pullulan-Based Antimicrobial Systems for Various Applications

**DOI:** 10.3390/ijms222413596

**Published:** 2021-12-18

**Authors:** Mahendra Rai, Magdalena Wypij, Avinash P. Ingle, Joanna Trzcińska-Wencel, Patrycja Golińska

**Affiliations:** 1 Department of Biotechnology, Sant Gadge Baba Amravati University, Amravati 444602, Maharashtra, India; 2 Faculty of Biological and Veterinary Sciences, Nicolaus Copernicus University, Lwowska 1, 87-100 Toruń, Poland; mwypij@umk.pl (M.W.); trzcinska@doktorant.umk.pl (J.T.-W.); 3 Biotechnology Centre, Department of Agricultural Botany, Dr. Panjabrao Deshmukh Krishi Vidyapeeth, Akola 444104, Maharashtra, India; ingleavinash14@gmail.com

**Keywords:** pullulan, exopolysaccharide, antimicrobial activity, drug delivery

## Abstract

Global reports on multidrug resistance (MDR) and life-threatening pathogens such as SARS-CoV-2 and *Candida cruris* have stimulated researchers to explore new antimicrobials that are eco-friendly and economically viable. In this context, biodegradable polymers such as nisin, chitin, and pullulan play an important role in solving the problem. Pullulan is an important edible, biocompatible, water-soluble polymer secreted by *Aureobasidium pullulans* that occurs ubiquitously. It consists of maltotriose units linked with α-1,6 glycosidic bonds and is classed as Generally Regarded as Safe (GRAS) by the Food and Drug Administration (FDA) in the USA. Pullulan is known for its antibacterial, antifungal, antiviral, and antitumor activities when incorporated with other additives such as antibiotics, drugs, nanoparticles, and so on. Considering the importance of its antimicrobial activities, this polymer can be used as a potential antimicrobial agent against various pathogenic microorganisms including the multidrug-resistant (MDR) pathogens. Moreover, pullulan has ability to synthesize biogenic silver nanoparticles (AgNPs), which are remarkably efficacious against pathogenic microbes. The pullulan-based nanocomposites can be applied for wound healing, food packaging, and also enhancing the shelf-life of fruits and vegetables. In this review, we have discussed biosynthesis of pullulan and its role as antibacterial, antiviral, and antifungal agent. Pullulan-based films impregnated with different antimicrobials such as AgNPs, chitosan, essential oils, and so on, forming nanocomposites have also been discussed as natural alternatives to combat the problems posed by pathogens.

## 1. Introduction

Alarming reports on multidrug resistance (MDR) in pathogens from various parts of the world have garnered the attention of researchers. Owing to the misuse and overuse of antibiotics, resistance in microbes is becoming a major threat to human health. MDR is mainly responsible for a high mortality rate, and expansive and enhanced treatment, and also affects the efficacy of antimicrobials [1]. Moreover, the new and emerging microbes like coronavirus (COVID-19) and *Candida auris* have also threatened the lives of the people, and therefore, there is a pressing need to search for effective antimicrobial drugs to tackle this problem. In this context, biodegradable polymers such as chitin, nisin, and pullulan can play a vital role as antimicrobials and also in the delivery of antimicrobial drugs. The extracellular secretion of this polymer was first reported in the fungus *Aureobasidium pullulans,* which occurs on diverse substrates [2,3,4]. Pullulan has excellent flexibility and solubility due to the alteration of 𝛼-1,4 and 𝛼-1,6 linkages [3]. It contains maltotriose units linked with α-1,6 glycosidic bonds [5], and it is an excellent agent for the formation of thin films because it is impermeable to oxygen, nonreducing, and not hygroscopic [6]. In addition, these thin films are transparent, tasteless, and odorless [7].

The basic advantage is that pullulan is classed as Generally Regarded as Safe (GRAS) by the Food and Drug Administration (FDA) in the USA, and therefore it has been used for the last two decades by food industries (including in food processing and food packaging), by cosmetics industries, and in biomedical applications [7,8,9]. 

Pullulan has already demonstrated remarkable antiviral, antitumor [10], and antibacterial activities [11,12]. It can also be used for the synthesis of capped AgNPs, which are also remarkable antimicrobial agents [13]. In addition, pullulan can be used for the delivery of antimicrobial drugs, essential oils, and nanomaterials of an antimicrobial nature [14,15]. Pullulan-based nanocomposite films are known to play a key role as a remarkable antimicrobial agent against various pathogens. For example, pullulan-based film impregnated with nisin, lauric arginate, and thymol forms an excellent composite that can be used as a potent antimicrobial agent against *E. coli*, *Staphylococcus aureus*, *Listeria monocytogenes*, and *Salmonella* spp. [16]. Such types of nanocomposite films can be applied in wound infections, particularly those caused by MDR bacteria and fungi. In addition, these nanocomposites can be used against foodborne pathogens, for increasing the shelf-life of fruits and vegetables, and also for food packaging purpose [12].

In this review, we have mainly focused on the biosynthesis of pullulan and its role as an antimicrobial agent. The pullulan-based films impregnated with different antimicrobials such as AgNPs, chitosan, essential oils, and so on, forming nanocomposites have also been discussed as eco-friendly and economically viable natural alternatives to combat the problems posed by MDR and emerging pathogens.

## 2. Pullulan: Biosynthesis and Production

**Figure 1:**
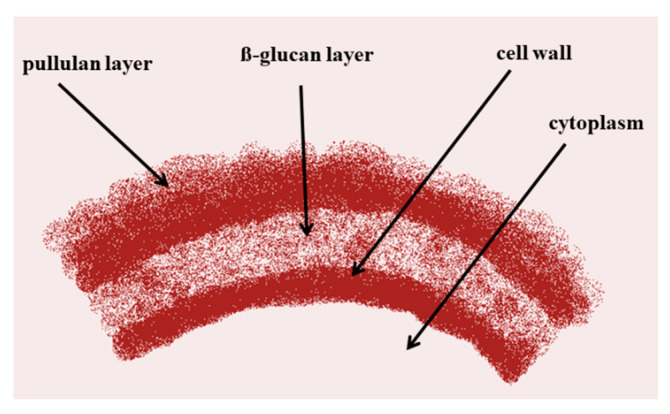
Extracellular synthesis of pullulan.

Microorganisms such as bacteria and fungi are well known for the production of extracellular metabolites (e.g., polysaccharides) [17]. The polysaccharides belong to the biopolymers and have diverse functional categories as intracellular polysaccharides, structural polysaccharides, and exopolysaccharides (EPS) [17,18]. The first report on pullulan production using yeasts belonging to the genus *Pullularia* came from the work of Bauer [4]. Later, Simon et al. [19] reported that pullulan forms a loose, slimy layer on the cell surface and is synthesized extracellularly at the cell wall membrane (Figure 1).

Recently, it has been suggested that pullulan biosynthesis takes place in the cell wall and periplasmic space of the *Aureobasidium melanogenum* P16 [20,21]. Pullulan is the main linear glucosic polysaccharide of fungal origin produced by *Aureobasidium pullulans* (de Bary) Arnaud (formerly known as *Pullularia pullulans* de Bary) Berkhout or *Dematium pullulans* (de Bary) [3]. Although synthesis of exopolysaccharide with high pullulan content is restricted to certain varieties of *A. pullulans* [22], other species belonging to the genus *Aureobasidium*, namely *A. melanogenum*, *A. leucospermi*, *A. proteae*, *A. thailandense,* and *A. nambiae* are also associated with pullulan production [21,23,24,25,26]. The capacity of pullulan production was also found in the saprophytic fungus *Tremella mesenterica* [27], parasitic fungal strains including *Teloschistes flavicans* [28] and *Cryphonectria parasitica* [29], edible mushrooms *Cyttaria harioti* and *Cyttaria darwinii* [30,31], yeasts such as *Rhodotorula bacarum* and *Rhodosporidium paludigenum* [32,33], and bacteria including *Micrococcus luteus* [34]. Overall, the microbial EPSs’ production, including pullulan, mostly depends on substrate composition (breeding factors), fermentation conditions (pH, temperature), and the improvement of *A. pullulans* strains [3,21,35]. The medium composition, especially sufficient amounts of nutrients with an optimized carbon/nitrogen ratio, enhances cell proliferation and increases metabolite formation [24,36]. *Aureobasidium pullulans* can use a wide variety of carbon sources for pullulan synthesis, as exemplified by media containing simple and complex sugars, such as glucose, fructose, mannose, maltose, as well as xylose, ribose, arabinose, sucrose, and lactose [37]. However, pullulan production is usually carried out in a medium containing a high concentration of sucrose, not glucose, as biosynthesis of this polymer is sensitive to glucose and the presence of a high glucose concentration in the medium represses many genes related to pullulan biosynthesis and decreases the yield of pullulan [38,39]. Therefore, glucose derepression is one way to enhance pullulan biosynthesis [21]. It has been reported that glucose-derepressed mutant of *A. melanogenum* with removed gene coding for CreA produced higher yield of pullulan. *CREA* or *MIG1* genes encoding for a glucose repressor, that is global regulator in fungi including yeasts, can play a crucial role in glucose repression. The Mig1 and CreA proteins, Cys_2_His_2_ (C_2_H_2_) zinc-finger proteins, are able to bind the well-characterized 5′-SYGGRG-3′-rich sites in the promoters of a variety of glucose-repressed genes, including those responsible for pullulan biosynthesis [39,40,41]. The glucose-derepressed mutant of *A. melanogenum* P16 produced more pullulan (64.93 g/L) from glucose (conc. 120.0 g/L) than the corresponding native strain (52.0 g/L) under the same conditions [39]. Other proteins such as Mig1 (the transcription factor), Hxk2 (hexose kinase), Reg1-Glc7 (protein kinase), Med8, Cyc8-Tup1, Snf1 (protein kinase), Adr1, and Cat8 are also involved in glucose repression and derepression [41]. Moreover, it is suggested that pullulan biosynthesis in *A. melanogenum* P16 is regulated by the Msn2 and the corresponding signaling pathway. The Msn2 is the C_2_H_2_ Zn finger protein and contains the DNA-binding domain which can recognize the specific promoter sequence (AGGGG and CCCCT) of the regulated genes, including most of the genes coding for pullulan biosynthesis [21]. The Msn2 protein localized in the nuclei of *A. melanogenum* P16 expresses the *UGP1* gene encoding for UDPG-pyrophosphorylase (Ugp1) catalyzing for UDP-glucose formation, the sole precursor of pullulan biosynthesis, and results in enhanced pullulan biosynthesis [21].

Singh and Saini [3] claimed that the ability to utilize different carbon sources was probably an individual characteristic of strains used for synthesis. As the rapid development of the biotechnology industry is related to the need to expand the raw material base with new, cheaper carbon sources [3,36], agricultural waste [42], olive oil and sucrose [43], deproteinized whey [44], beet molasses [45], sweet potato [46], and potato starch hydrolysate [47] have been utilized as low-cost substrates for efficient production of pullulan through fermentation process by using *A. pullulans* [48]. Likewise, each strain has its own preferences for the source of nitrogen, which should be present in the culture medium for the production of pullulan [24]. Most of the used strains preferred ammonium sulfate [3], but other nitrogen sources in the fermentation medium such as soybean hydrolyzate, peptone or tryptone, ammonium acetate, and sodium nitrate can also be utilized for the biosynthesis and secretion of pullulan [36].

**Figure 2:**
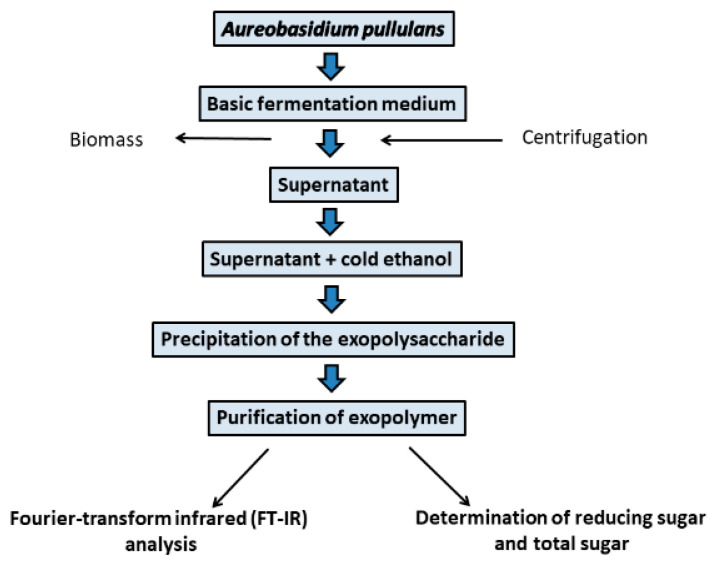
General approach for pullulan biosynthesis by *Aureobasidium pullulans*.

Moreover, pH of the culture medium is the decisive factor influencing the morphology of fungal strains and the efficiency of pullulan production [3,36]. *Aureobasidium pullulans* is a polymorphic fungus that can form the budding blastoconidia (yeast-like cells), mycelia, colorless chlamydospores divided by septa, and ones that secret black melanin. To date, chlamydospores and unicellular blastoconidia are known to produce pullulan [3,21,49]. Optimal initial pH for the production of pullulan is in the range of 5.5–6.5 pH [50]. The optimum temperature for the synthesis of pullulan varies slightly for different strains of *A. pullulans* and has been reported in the range of 25–28 °C [3,36]. The important aspect of pullulan synthesis in industry is the use of *A. pullulans* strains with the ability to inhibit the synthesis of melanin compounds [24]. Kachhawa and coauthors [51] reported that melanin was one of the obstacles of pullulan production in their study, and this compound was removed using a suitable solvent during the fermentation and precipitation of EPS. A typical approach for pullulan synthesis from *Aureobasidium* spp. strains is given in Figure 2.

Until 2020, the mechanism of pullulan synthesis had not been well elucidated, and it has taken a long time to explain which enzymes and encoding genes are involved in the synthetic pathways of this EPS [21]. It is well known that UDP-glucose is a precursor of pullulan biosynthesis [21,52]. However, Shingel [53] also suggested that glucose-containing lipid intermediates may be involved in this pathway. Later, Duan et al. [37] reported that the presence of key enzymes such as α-phosphoglucose mutase, uridine diphosphoglucose (UDPG) pyrophosphorylase, and glucosyltransferase are involved in conversion of glucose to pullulan molecules. On the other hand, Cheng and coauthors [24] reported that hexokinase and isomerase are needed to convert different carbon sources into the pullulan precursor, UDPG. In addition, another gene, namely *UGT1*, encoding for a UDP-glucose:glycoprotein glucosyltransferase-like protein (Ugt1), has also been described as implicated with pullulan synthesis [52]. Deletion of this gene from *A. melanogenum* P16 highly reduced yield of biosynthesized pullulan, while its overexpression caused pullulan overproduction (from 63.38 g/L in native strain to 80.2 g/L in a modified one). It was suggested that Ugt1 protein might function as a “folding sensor” for incompletely glucosylating folder glycoproteins [54]. Recently, it was found that pullulan synthetase encoded by *PUL1* gene could be an auxiliary protein for one component required for the α-glucan synthetase 2 (AmAgs2) activity that is a key enzyme for pullulan biosynthesis. The deletion of the *PUL1* gene resulted in no pullulan synthesis in two mutants of *A. pullulans* [21,54].

## 3. Antimicrobial Potential of Pullulan-Mediated Bionanoparticles

It is well established that various metal nanoparticles find tremendous applications in different sectors, including health care and biomedicine. Among these, gold, silver, zinc, copper, and so on are attracting a great deal of attention because of their widespread usage. However, considering their toxicological concerns, in recent times, a new “green” concept has been introduced known as biosynthesis of metal nanoparticles. To date, a number of biopolymers have been extensively used for the synthesis of nanoparticles (e.g., chitosan, dextran, gelatin, sodium alginate, albumin) [55]. Recently, however, scientists have been mainly focusing on the usage of pullulan, an exopolysaccharide secreted by fungi (e.g., *Aureobasidium pullulans*) as both a reducing and stabilizing agent. As discussed earlier, pullulan is a microbial linear homopolysaccharide consisting of repeating units of glucose that is often referred to as a biopolymer of α-1,6 glycosidic-linked maltotriose units [56,57]. Due to its promising adhesive and film-forming properties, pullulan has been used to make compression moldings, fibers, drug delivery carrier materials, and edible films [9,57,58,59,60].

Pullulan-based nanoparticles are reported to have efficient antibacterial activities against a variety of pathogens. For example, Ganduri et al. [55] developed a rapid method for the biosynthesis of pullulan-stabilized silver nanoparticles (AgNPs) using pullulan as the reducing and stabilizing agent. The nanoparticles thus synthesized were found to be spherical in shape and had an average size between 10 and 55 nm. Further, these pullulan-stabilized AgNPs were evaluated for their antibacterial efficacy against two Gram-positive (*Bacillus subtilis* and *Staphylococcus aureus*) and two Gram-negative (*Escherichia coli* and *Serratia marcescens*) bacterial pathogens using the agar well diffusion method. The results obtained revealed that all the bacterial pathogens were inhibited in a dose-dependent manner. However, the inhibitory effect of pullulan-stabilized nanoparticles against tested strains decreased as follows: *B. subtilis*, *S. aureus*, *S. marescenes*, and *E. coli* [55]. 

In addition to common pathogens, pullulan-mediated nanoparticles demonstrated significant activity against foodborne and multidrug-resistant pathogens. In a study, Kanmani and Lim [61] synthesized AgNPs using pullulan biopolymer and evaluated them in vitro for antibacterial, antifungal, and antibiofilm activities. These nanoparticles were rod-like and hexagonal in shape and had a size of 2–40 nm. Their antibacterial activity was examined against foodborne and multidrug-resistant pathogens such as *E. coli*, *Klebsiella pneumoniae*, *Pseudomonas aeruginosa*, and *L. monocytogenes*; their antifungal activity against *Aspergillus* spp. and *Penicillium* spp.; and their antibiofilm activity against biofilm-forming pathogens such as *E. coli*, *Bacillus cereus*, *L. monocytogenes*, and *P. aeruginosa*. The observations recorded revealed that among the bacterial pathogens, *P. aeruginosa* was found to be most susceptible to AgNPs, followed by *K. pneumoniae* and *E. coli*. On the contrary, food-born pathogen *L. monocytogenes* was reported to be less susceptible to biogenic AgNPs. As far as antifungal activity is concerned, both the fungal pathogens were found to be susceptible to AgNPs in a dose-dependent manner. Moreover, promising activity was also reported against all the biofilm-forming bacteria [61].

Similarly, an attempt has been made towards the synthesis of AgNPs using pullulan produced from a fungus, *Aureobasidium mangrovei*, isolated from Oman. Moreover, antibacterial and antifungal activities of thus synthesized pullulan-AgNPs were evaluated against *E. coli*, *S. aureus*, *B.* cereus, *P. aeruginosa*, and fungi like *Curvularia lunata*, *Fusarium incarnatum*, *Aspergillus niger*, *A. flavus*, *A. ochraceus*, and *Penicillium* sp. It was observed that these pullulan-mediated AgNPs were highly effective in the growth inhibition of all the tested bacteria and fungi [62]. Overall, higher susceptibility of Gram-negative bacteria compared to Gram-positive bacteria to pullulan-capped AgNPs, as showed by Kanmani and Lim [61] and others [63], is explained by differences in their cell structure, because Gram-positive bacteria are composed of a thick three-dimensional peptidoglycan layer compared to that of Gram-negative bacteria. The peptidoglycan layer is a complex structure consisting of linear polysaccharide chains cross-linked by shorter peptides which hinder the penetration of AgNPs in Gram-positive bacteria [64]. However, results of the antibacterial activity of pullulan-stabilized nanoparticles by Kanmani and Lim [61] are in contradiction to those by Ganduri et al. [55]. The main reason for the difference in activity of AgNPs on both types of bacteria may be due to the physical and chemical properties of nanoparticles, including the size, shape, and surface charge [65,66]. Pal et al. [67] when comparing spherical, rod-shaped, and truncated triangular AgNPs, showed that the truncated triangular AgNPs revealed the best antimicrobial activity. More recently, however, Cheon et al. [68] reported that antimicrobial activity obtained from the inhibition zone was in the order of spherical > disk > triangular plate AgNPs. The pullulan-stabilized AgNPs obtained in both studies cited above were also different in shape [55,61]. In the case of the antimicrobial activity of AgNPs, the Ag ions (Ag^+^) are released from the NPs (Ag°). Therefore, the amount of Ag ions released from nanoparticles depends on the surface area, which results from the shape of the NPs [68]. Overall, pullulan is considered as a biopolymer material for the synthesis of silver nanoparticles to improve the reduction process without accelerating, reducing, or complexing agents. Moreover, the structure of the polysaccharides in pullulan is reported to enhance the antibacterial activity by encapsulating the Ag-NPs, thus producing more stable, uniform, and monodisperse nanoparticles. In addition, the particle size can be tailored to the desired size with the incorporation of capping agents such as polymers, including pullulan [69]. It was observed that initially, gamma irradiation reduced the silver ions, and pullulan polymer acts as a capping and stabilizing agent, leading to the formation of stabilized pullulan-capped AgNPs [69]. The capping of AgNPs stabilizes the nanoparticles and avoids the possibility of agglomeration. Moreover, surface-capped AgNPs demonstrate better antimicrobial activity after capping [70,71].

**Figure 3:**
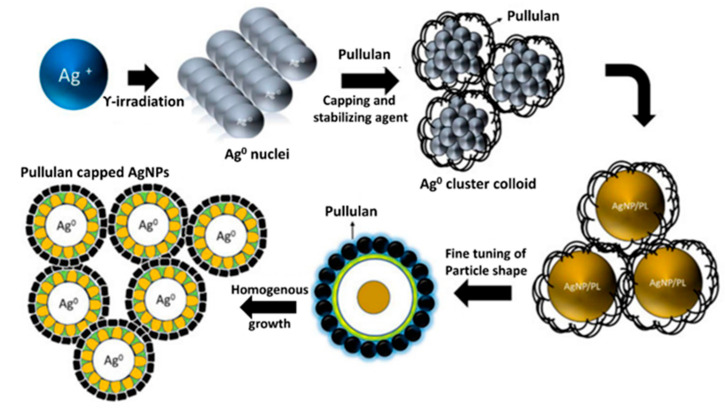
Schematic representation of mechanism for the synthesis and growth of AgNPs on pullulan using gamma irradiation (adapted from [69]; an open access article).

Figure 3 shows a schematic representation of the mechanism for the synthesis and growth of AgNPs on pullulan using gamma irradiation.

**Figure 4:**
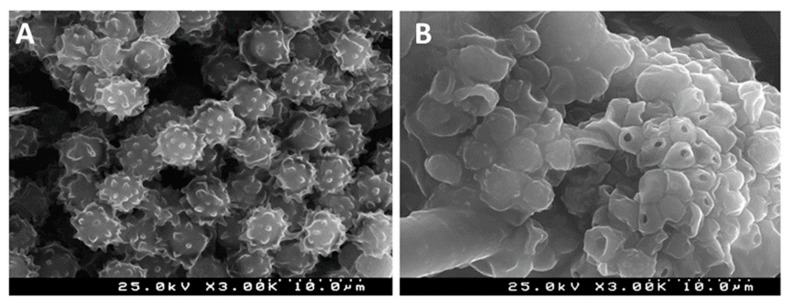
SEM images of conidia of *A. niger*: (**A**) after treatment with neat pullulan film (control); (**B**) after exposure to pullulan nanocomposite film (experimental) (adapted from [15]; with copyright permission from Elsevier).

The surface charge of these pullulan-stabilized AgNPs was found to be −72 mV, which made them highly stable with a low tendency for aggregation. Further, it was also reported that these nanoparticles exhibit strong antibacterial activity against *S. aureus* [69]. Similarly, Pinto et al. [15] developed a transparent nanocomposite thin film of pullulan and AgNPs and evaluated its activity against *A. niger*. The scanning electron microscopic study of conidia of *A. niger* after the treatment with this pullulan nanocomposite film clearly showed the disruption effects on conidia after this interaction (Figure 4). These findings suggest that the conidial damage may be associated with cell wall disruption and subsequent leaking of the cytoplasmic content, thus causing cell death. Moreover, it was reported that the cell disruption effect depends on the concentration of AgNPs in the film—the higher the concentration, the higher the cell disruption observed. 

All the above studies strongly show the promising antimicrobial potential of pullulan-based nanoparticles against a wide range of microbial pathogens including multidrug-resistant and biofilm-forming organisms. 

## 4. Pullulan-Based Antimicrobial Nanocomposites 

The non-biodegradable synthetic polymers used in food packaging pose a serious concern for the environment and also to human health. Therefore, special focus has been applied to the application of eco-friendly and biodegradable packaging materials, mostly obtained from natural polymers, for food packaging. In this context, researchers believe that pullulan can be a suitable polymer for this purpose due to its water-soluble, non-toxic, and non-mutagenic edible nature. Most importantly, this biopolymer has excellent film-forming and adhesive properties, and hence it can be used in the development of edible films or coatings for different food products [7]. 

Recently, the development of edible films containing antimicrobial compounds has been attracting a great deal of attention all over the world as an effective treatment to extend the shelf life of fruits and vegetables and to reduce the risk of pathogens. For example, in one study the authors demonstrated the antimicrobial potential of pullulan films, incorporating AgNPs, zinc oxide nanoparticles (ZnONPs), oregano oil (2%), and rosemary oil (2%), against *L. monocytogenes* and *S. aureus* responsible for spoilage of Turkey deli meat quality [72]. The results showed that *S. aureus* was more susceptible than *L. monocytogenes* to the edible pullulan films containing the above nanoparticles and essential oils. In another study, Wu and Chen [57] prepared pullulan-based coatings by combining anti-browning and antibacterial agents. Pullulan was used as a thickener that can form semipermeable films, glutathione was used as an effective reducing agent, and chito-oligosaccharide was used as an antibacterial agent (e.g., 1% pullulan; 0.8% glutathione + 1% chito-oligosaccharides; and 0.8% glutathione + 1% chito-oligosaccharides + 1% pullulan). Moreover, the efficacy of these edible coatings was evaluated on apple slices during hypothermic storage. It was observed that the pullulan coatings effectively inhibited microbial growth and the respiration rate of apple slices during hypothermic storage compared with the control. In addition, the application of these pullulan-based coatings was also found to retard enzymatic browning, maintain firmness, and decrease weight loss. These findings suggest that application of pullulan-based coatings in combination with glutathione and chito-oligosaccharides can be helpful to extend the shelf life of apple slices [57]. In addition to fruits, pullulan-based edible coatings can be effectively used to increase the shelf-life of eggs. Morsy et al. [73] coated fresh eggs with pullulan and pullulan-containing nisin, using non-coated eggs as control. These eggs were kept for 10 weeks at 25 and 4 °C. The observations recorded suggested that eggs coated with pullulan remain healthy in terms of lower weight loss and yolk index compared to non-coated eggs and eggs coated with pullulan-containing nisin. From the study, authors concluded that pullulan coatings can preserve the internal quality, prolong the shelf-life, and minimize the weight loss of fresh eggs [73].

Moreover, different pullulan-based composites used in combination with other polymers, such as pectin and chitosan, for the development of films and coatings for food preservation and packaging are gaining considerable importance. Priyadarshi et al. [74] developed edible films by mixing pectin and pullulan in different ratios and evaluated their efficacy in food packaging applications. The formation of an intermolecular H-bond was observed after characterization with FTIR between the hydroxyl group of pullulan and the carboxyl group of pectin, which makes the complex stronger. It was observed that film prepared with a 50:50 blend ratio of pullulan and pectin exhibited the highest thermal stability and surface hydrophobicity. Besides, this blending was also found to increase the strength while maintaining flexibility and stiffness compared to the individual films. 

In another study, authors developed pullulan–chitosan and pullulan–carboxymethyl chitosan (CMCH) blended films and investigated their viscosity, mechanical properties, barrier properties (water and oxygen), water solubility, and so on. It was reported that the addition of the chitosan or CMCH to pullulan significantly modified the mechanical and oxygen barrier properties of the film. The blend ratio of pullulan and chitosan to achieve films with optimum mechanical properties and oxygen and water barrier properties was found to be 1:1. Application of such films in food packaging can promisingly increase the shelf-life of food products [56,57]. Similarly, Li et al. [75] studied the effect of pH on the efficacy and properties of pullulan–chitosan blended films. It was reported that the extended conformation of chitosan in a pH 4.0 solution increased the intermolecular interactions with pullulan compared to the more compact coiled form, resulting in higher tensile strength and barrier properties of the pullulan–chitosan film and higher viscosity of the film-forming solution. All these studies confirmed that application of pullulan-based edible coatings or films are significantly helpful to maintain the quality of fruits and food products by protecting their spoilage from microbial pathogens and therefore can also be used in food packaging. 

Pullulan-based smart packaging impregnated with nanoparticles and essential oils are gaining importance nowadays due to their promising antimicrobial applications in different sectors. Recently, an article focusing on the application of pullulan active packaging incorporated with AgNPs and essential oils in reducing meat perishability has been published [76]. In this article, the authors reviewed the promising role of pullulan active packaging incorporated with different nanoparticles and essential oils. It was proposed that these active packages are highly effective in the growth inhibition of *Salmonella typhimurium*, *S. aureus*, *E. coli*, and *Clostridium perfringens*, which are responsible for outbreaks in meat through the negation of cholesterol oxidation products. Pullulan active packaging can effectively manage all such pathogens and considerably increase the shelf life of, and consumer demands for, meat [76].

There are reports that emphasize the application of essential oils in combination with nanomaterials for the preparation of edible films that can be potentially used to improve the microbiological safety of foods. Morsy et al. [77] demonstrated the efficacy of pullulan films containing essential oils and nanoparticles against four foodborne pathogens. The initial findings suggested that 2% oregano essential oil was active against *S. aureus* and *S. typhimurium*; however, it was not significantly effective against *L. monocytogenes* and *E. coli* O157:H7. On the other hand, 2% rosemary essential oil was found to be effective against all four above-mentioned pathogens when compared with 1%. Similarly, their combination with zinc oxide and silver nanoparticles were also found to be much more efficient against the tested pathogens. The results revealed that edible films made from pullulan and incorporated with essential oils or nanoparticles may improve the safety of refrigerated, fresh, or further processed meat and poultry products. Similarly, Gniewosz et al. [14] developed pullulan film containing caraway essential oil and evaluated its antimicrobial efficacy against foodborne pathogens including *Salmonella enteritidis*, *S. aureus*, *Saccharomyces cerevisiae*, and *A. niger,* and its effects on quality in fresh baby carrot. It was observed that caraway essential oil significantly inhibited the growth of all the tested microorganisms. The pullulan films containing 8% to 10% caraway essential oil were found to be most effective against all these food pathogens. In addition, it was also recorded that fresh baby carrot treated with pullulan–caraway oil coating maintained better visual acceptability than that of non-treated samples. Hence, it can be concluded that application of a pullulan film with incorporated caraway essential oil can extend the microbiological stability of minimally processed foods.

More recently, Luís et al. [9] developed pullulan–apple fiber biocomposite films and used them as a novel food packaging material. After evaluation of optical, mechanical, and barrier properties of thus developed pullulan-based biocomposite films, it was reported that these films possess significantly higher tensile index and elastic modulus compared to normal pullulan films. Moreover, other properties such as water vapor permeability, water vapor transmission rate, and water contact angle were also found to be higher. In addition, these films showed antibacterial activity against seven foodborne pathogens: *S. aureus* (ATCC 25923), *L. monocytogenes* (LMG 16779), *Enterococcus faecalis* (ATCC 29212), *B. cereus* (ATCC 11778), *S. typhimurium* (ATCC 13311), *E. coli* (ATCC 25922), and *P. aeruginosa* (ATCC 27853).

## 5. Pullulan-Based Nanomaterials in Delivery of Biomolecules/Drugs

**Figure 5:**
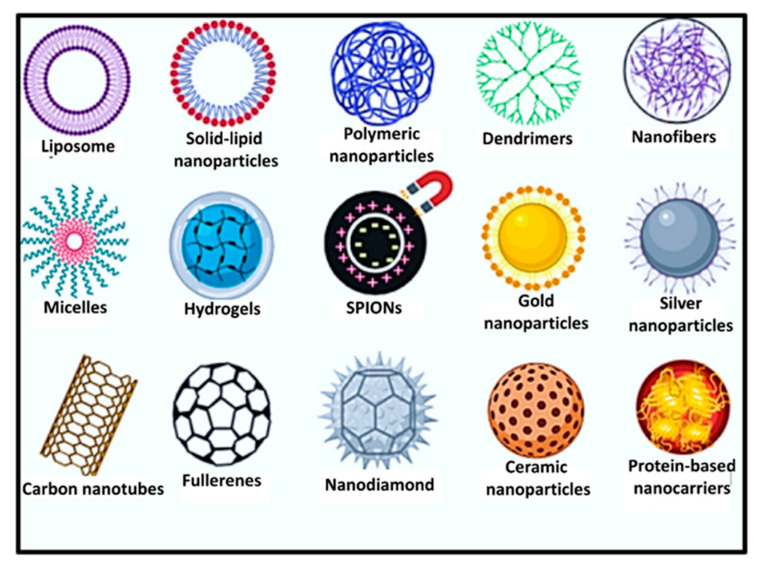
Pullulan-based nano-drug delivery systems (adapted from [78] with copyright permission from Elsevier).

Nowadays, drug delivery systems are reported to play a promising role in the administration of different drugs due to their sustained release property. The nanoplatforms designed for sustained release of drugs release drug molecules at a predetermined rate by maintaining a constant drug level for a specific period with minimum side effects. Raychaudhuri et al. [78] classified different pullulan-based nano-drug delivery systems as lipid-based systems (e.g., nanostructured lipid carriers, solid lipid nanoparticles, cubosomes, microemulsion, liposomes, etc.); polymer-based nanosystems (dendrimers, polymeric nanocarrier micelles, hydrogels); metallic nanocarriers (e.g., superparamagnetic nanocarriers, silver- and gold-based nanocarriers); and carbon-based nanoplatforms (fullerenes, carbon nanotubes, nanodiamond, etc.) (Figure 5). 

In the context of drug delivery, transmucosal delivery is the first-line option for the systemic delivery of many drugs, including proteins, drug molecules with therapeutic potential, and so on. However, this strategy requires developing suitable and appropriate carriers, because such biomolecules (e.g., proteins) are very sensitive, and can undergo degradation very fast due to pH and enzymatic occurrences. Recently, however, different polymeric nanoparticles have been shown to play an important role in overcoming these challenges through the development of adequate carrier designs, offering an appealing number of properties required for transmucosal delivery.

Grenha and Rodrigues [79] opined that pullulan-based nanoparticles can be most suitable for future therapeutic applications in transmucosal protein delivery. In addition, several other studies have demonstrated the use of polymeric nanoparticles as a carrier system in the delivery of a variety of components like proteins, drugs, and so on. Polysaccharides like pullulan are favorably used for the production of such carriers due to their unique properties, such as their structural flexibility and propensity to evidence biocompatibility and biodegradability. For example, Dionísio et al. [80] synthesized pullulan-based nanoparticles by a mild method of polyelectrolyte complexation using pullulan as a neutral polysaccharide. Further such pullulan-based nanoparticles were used as carrier for the delivery of transmucosal protein. Bovine serum albumin (BSA) was used as a model protein. These nanoparticles have been found to produce a slow and steady release of BSA. Therefore, it is believed that due to the bio-adhesiveness of pullulan and pullulan-based nanoparticles, these hold potential for application in transmucosal protein delivery, with a particular focus on the nasal and pulmonary routes.

Apart from delivery of macromolecules, pullulan-based nanocomposites are also found to have application in the delivery of nasal vaccines. Nasal immunization using different nanoparticles has shown promising results. Cevher et al. [81] prepared nanocomposites containing BSA for nasal vaccination by using an electrostatic interaction process between polycation N-trimethyl chitosan chloride, chitosan glutamate, chitosan chloride, and polyanion carboxymethyl pullulan. The results revealed that these nanocomposites can potentially be used as nanoparticulate carriers for nasal vaccination.

## 6. Conclusions

The worldwide increase of multidrug resistance and the emergence of new microbial pathogens such as *Candida auris* and COVID-19 have encouraged the scientific community to search for alternatives to existing antimicrobial and drug-delivery systems, such as biodegradable polymers including nisin, chitin, and pullulan. The latter is not only biodegradable, but also characteristically biocompatible, water-soluble, edible, and classed as Generally Recognized as Safe (GRAS). Pullulan is mainly synthesized by *Aureobasidium pullulans*, which is characterized by maltotriose units linked with α-1,6 glycosidic bonds. However, there are some other *Aureobasidium* species, including *A. melanogenum*, *A. leucospermi*, *A. proteae*, *A. thailandense*, and *A. nambia*, that are known for pullulan production.

The pullulan-based nanoparticles have already demonstrated their potential against various Gram-positive and Gram-negative human pathogenic bacteria and fungi. They are also effective against multidrug-resistant and biofilm-forming bacteria. Pullulan is an efficient synthesizer of antimicrobial AgNPs and also acts as a capping agent. In smart food packaging, biodegradable materials play a vital role, and in this context, pullulan-based edible coatings for food packaging are a boon, being a non-toxic, edible, eco-friendly, economically viable, and efficient antimicrobial agent. The pullulan-based nanocomposites containing AgNPs, essential oils, or other antimicrobial drugs are remarkably efficacious against foodborne bacteria. Finally, an important area that warrants further investigation is the use of pullulan-based antimicrobial systems in the delivery of bioactive nature-based secondary metabolites, essential oils, antimicrobial agents, and drugs. 

## Data Availability

No new data were created or analyzed in this study. Data sharing is not applicable to this article.
